# Mutations in POLE and survival of colorectal cancer patients – link to disease stage and treatment

**DOI:** 10.1002/cam4.305

**Published:** 2014-08-01

**Authors:** Albrecht Stenzinger, Nicole Pfarr, Volker Endris, Roland Penzel, Lina Jansen, Thomas Wolf, Esther Herpel, Arne Warth, Frederick Klauschen, Matthias Kloor, Wilfried Roth, Hendrik Bläker, Jenny Chang-Claude, Hermann Brenner, Michael Hoffmeister, Wilko Weichert

**Affiliations:** 1 Institute of Pathology, Heidelberg University HospitalGermany; 2 Division of Clinical Epidemiology and Aging Research, German Cancer Research Center (DKFZ)Heidelberg, Germany; 3 German Consortium for Translational Cancer Research (DKTK)Germany; 4 Institute of Pathology, Charité University MedicineBerlin, Germany; 5 Department of Applied Tumor Biology, Institute of Pathology, University of HeidelbergHeidelberg, Germany; 6 Unit of Genetic Epidemiology, German Cancer Research Center (DKFZ)Heidelberg, Germany; 7 National Center for Tumor Diseases (NCT)Heidelberg, Germany

**Keywords:** Colorectal cancer, DACHS, MSS, mutation, POLE, polymerase *ε*, ultramutator phenotype

## Abstract

Recent molecular profiling studies reported a new class of ultramutated colorectal cancers (CRCs), which are caused by exonuclease domain mutations (EDMs) in DNA polymerase *ε* (POLE). Data on the clinical implications of these findings as to whether these mutations define a unique CRC entity with distinct clinical outcome are lacking. We performed Sanger sequencing of the POLE exonuclease domain in 431 well-characterized patients with microsatellite stable (MSS) CRCs of a population-based patient cohort. Mutation data were analyzed for associations with major epidemiological, clinical, genetic, and pathological parameters including overall survival (OS) and disease-specific survival (DSS). In 373 of 431 MSS CRC, all exons of the exonuclease domain were analyzable. Fifty-four mutations were identified in 46 of these samples (12.3%). Besides already reported EDMs, we detected many new mutations in exons 13 and 14 (corresponding to amino acids 410–491) as well as in exon 9 and exon 11 (corresponding to aa 268–303 and aa 341–369). However, we did not see any significant associations of EDMs with clinicopathological parameters, including sex, age, tumor location and tumor stage, CIMP, KRAS, and BRAF mutations. While with a median follow-up time of 5.0 years, survival analysis of the whole cohort revealed nonsignificantly different adjusted hazard ratios (HRs) of 1.35 (95% CI: 0.82–2.25) and 1.44 (0.81–2.58) for OS and DSS indicating slightly impaired survival of patients with EDMs, subgroup analysis for patients with stage III/IV disease receiving chemotherapy revealed a statistically significantly increased adjusted HR (1.87; 95%CI: 1.02–3.44). In conclusion, POLE EDMs do not appear to define an entirely new clinically distinct disease entity in CRC but may have prognostic or predictive implications in CRC subgroups, whose significance remains to be investigated in future studies.

## Introduction

Colorectal cancer (CRC) is the third most common cancer in men and the second most common cancer in women worldwide. For 2008, more than 1 million new cases and ∼600,000 deaths have been estimated which makes CRC the fourth leading cause of death from cancer among adults [1].

CRC is a genomic disease that can be inherited but mostly arises sporadically and comprises several molecular subtypes associated with different biological and clinical behavior [2]. The common driver of tumor development is genomic instability of which chromosomal instability (CIN) is by far the most prevalent disease causing mechanism, two-thirds of all cases have been attributed to CIN [3, 4]. About 10–15% of CRCs arise through loss of function of DNA mismatch repair (MMR) genes leading to an inability to correct base mismatches, as well as insertions and deletions during DNA replication at repetitive sequences (microsatellite instability, MSI) resulting in a hypermutation phenotype. Patients with high-frequency MSI follow a distinct clinical cause with significantly improved prognosis compared to microsatellite stable (MSS) tumors and potential differences in the response to chemotherapeutic agents [5]. Besides these two major molecular types of CRC, two other well-defined alternative routes for CRC development have been described as follows: homozygous germline inactivation of the base excision repair gene mutY homologue (MUTYH) leading to a polyposis phenotype and the concomitant methylation of many gene loci resulting in the CpG island methylator phenotype (CIMP) [6]. However, some overlap exists between these major disease mechanisms, for example, hypermethylation can affect the MMR gene MLH1 with a MSI-high phenotype and is then frequently associated with mutations in the BRAF gene mainly affecting codon 600 of the corresponding protein [7].

Very recently, four independent studies [8–11] reported both germline and sporadic mutations in the exonuclease domain (EDM) of DNA polymerase *ɛ* (POLE) in a small subset of CRC, which interfere with the proofreading ability of the enzyme leading to a misincorporation of bases in the daughter strand during DNA replication [12–15]. Investigating familial CRC cases by whole genome sequencing and loss of heterozygosity analysis, Palles et al. [8] showed germline POLE (and also polymerase delta [POLD]) mutations to confer high penetrance predisposition to multiple adenomas and the occurrence of multiple CRC, thereby pointing to a new molecularly well-defined CRC syndrome. Additionally, using exome sequencing approaches, the TCGA consortium [9] and Seshagiri and colleagues [10] reported on a small subgroup of MSS CRC with very high mutational rates, exceeding 50 mutations per megabase. While looking for potential molecular mechanisms driving genomic instability in these tumors, they identified recurrent somatic missense mutations in POLE as a likely cause. Their suggestion of a causal role of POLE mutations in the constitution of an ultramutator phenotype CRC was backed by previous observations in mice being homozygous for a mutation in POLE that inactivates exonuclease activity. These mice displayed high mutation rates accompanied by increased frequencies of colorectal adenomas and carcinomas [16].

Taken together, these findings strongly argue for a novel biological subtype of CRC, which directly raises the clinically relevant question, whether this subgroup of CRC, like MSI, also constitutes a recognizable distinct clinicopathological disease entity with a distinct patient outcome.

Hence, we investigated the type and frequency of POLE mutations in patients with MSS CRC of a well characterized population-based patient cohort study and analyzed the associations between the mutation status and all major CRC-related epidemiological, pathological, genetic and clinical parameters, including overall survival (OS) and disease-specific survival (DSS).

## Patients and Methods

### Study design and study population

The cohort is derived from a large ongoing population-based case–control study in southwestern Germany (DACHS: Darmkrebs: Chancen der Verhütung durch Screening [colorectal cancer: potentials of prevention through screening]) with extensive follow-up data of enrolled patients. Details of the study design, participation rates and follow-up have been reported previously [17–19]. Briefly, patients aged 30 or older with a histologically confirmed first diagnosis of primary CRC, who were physically and mentally able to participate and to communicate in German, were recruited in all 22 hospitals of the study region offering CRC surgery. Community-based control subjects were randomly selected from population registries and frequency matched to cases with respect to age, sex and county of residence. Controls with a history of CRC were excluded; otherwise inclusion and exclusion criteria were the same as in cases. Participants with hereditary CRC syndromes were not excluded. In this study, only patients with follow-up information and available tumor tissue were analyzed. The study was approved by the ethics committees of the Medical Faculty at the University of Heidelberg and of the Medical Chambers of Baden-Wuerttemberg and Rhineland-Palatinate. Written informed consent including the analysis of tumor tissue from patients with CRC was obtained from each participant.

### Data collection and follow-up

As reported previously [17–20], patients provided information in a face-to-face interview conducted by trained interviewers. Additionally, discharge letters and pathology reports were gathered. On average 3 years after diagnosis, a questionnaire was sent to the treating physicians to collect information on cancer-related therapy, intermittent diagnoses of concomitant diseases and potential CRC recurrence.

About 5 years after diagnosis, additional information was collected from the patients alive, including newly diagnosed diseases and recurrences, which were corroborated by medical records. For those alive at 3-year but not at 5-year follow-up information about recurrent disease was requested from the physicians. Data on vital status and date of death were obtained from the population registries. Causes of death were corroborated by death certificates obtained from the health authorities in the Rhine–Neckar region and coded according to WHO standards.

Follow-up time was calculated as the time between the date of diagnosis and the date of event or censoring. Follow-up time of patients without any event of interest (death, recurrence) was censored at the date of the last follow-up or on 31 December 2012, whichever was first.

### Cohort characteristics and tissue processing of tumor samples

Formalin-fixed paraffin-embedded (FFPE) samples of CRC were collected from the pathology departments of the cooperating clinics and transferred to the tissue bank of the National Center for Tumor Diseases (NCT) in Heidelberg. For this study, we used all CRC cases serviced at the University Hospital Heidelberg with a MSS phenotype (*n* = 431). MSS was determined as described previously [18]. For the analysis of *POLE*, 56 cases were excluded from sequencing due to poor DNA quality so as to 373 samples were processed for mutation analysis. For 368 (of 373) sequenced cases, detailed clinical data were available for statistical analysis.

### DNA extraction and sequencing

CRC cases were analyzed for mutations in exons 9, 11, 13, and 14 of *POLE* (NM.006231) by Sanger sequencing. DNA was isolated from a microdissected section of a tumor tissue block from areas where a high-tumor cell concentration (at least 70% tumor cell content) had been microscopically identified by a board certified pathologist. DNA isolation was performed using a commercial DNA extraction kit (DNeasy, Qiagen, Hilden, Germany) according to the manufacturer's protocol. Exons 9, 11, 13, and 14 were amplified with the following primers: 5′-gtgttcagggaggcctaatg-3′ (exon 9 forward), 5′-gggcagatgctgctgtagta-3′ (exon 9 reverse), 5′-actttgggagaggaatttgg-3′ (exon 11 forward), 5′-cctaagtcgacatgggaagc-3′ (exon 11 reverse); 5′-catcctggcttctgttctca-3′ (exon 13 forward), 5′-gagcgggctggcatacat-3′ (exon 13 reverse), 5′-accctgggctcttgattttt-3′ (exon 14 forward), and 5′-cacctccattcagctccagt-3′ (exon 14 reverse). Bidirectional Sanger sequencing of all PCR products was subsequently conducted on an ABI 3500 Genetic Analyzer (Life Technologies, Carlsbad, CA) using the BigDye Terminator v1.1 Cycle Sequencing Kit (Life Technologies) according to standard protocols.

### In silico analysis of mutations

The biological impact of the mutations on the structure and function of the respective protein product was predicted in silico by the use of four different software tools: Provean (http://provean.jcvi.org/index.php) [21], Sift (http://sift.jcvi.org/) [22], MutationTaster (http://www.mutationtaster.org/) [23] and PolyPhen (http://genetics.bwh.harvard.edu/pph/data/) [24]. Additionally, we used the COSMIC (catalogue of somatic mutations in cancer) to check our sequencing data for mutations that have already been reported elsewhere.

### Statistical analysis

We first described clinical, pathological and behavioral characteristics of the patients according to their POLE mutation status. Using Cox proportional hazards regression models, we estimated crude and adjusted hazard ratios and their 95% confidence intervals of the association of POLE mutation and OS. In the adjusted analyses, we included age at diagnosis, sex, stage at diagnosis, location of the tumor (proximal colon (from coecum to transversal colon), distal colon (from left flexure to sigmoid), and rectum [including rectosigmoid]), chemotherapy and neoadjuvant treatment as covariates, and accounted for late entry, that is, the potentially delayed time period between date of diagnosis and date of enrolment. Additional stratified analyses were performed by age, gender, stage, grade, location, and by treatment with chemotherapy.

Direct adjusted survival curves were generated to illustrate the association of POLE mutation and OS. Unlike unadjusted Kaplan–Meier curves, the adjusted survival curves take potential effects of covariates into account as included in the multivariate Cox models [25].

The main analyses were repeated to investigate potential associations with CRC survival only. All analyses were performed with SAS, software version 9.2 (SAS Institute, Cary, NC). Tests for statistical significance were two-sided and defined by *P* < 0.05.

## Results

### *POLE* mutations in MSS CRC

Patients in this cohort were diagnosed between 2003 and 2006 and followed up for a median time of 5.0 years. End of follow-up was due to censoring, death, or until 31 December 2012. Of 368 patients, 140 (38%) were female and 228 (62%) were male. The mean age was 68 years. The majority of cases were diagnosed with tumor stage II (118, 32%) or III (129, 35%) while the remaining cases were fairly evenly distributed between stages I and IV. Forty-four percentage of the tumors were located in the rectum, 29% located in the distal, and 27% in the proximal colon. A total of 52 (14%) patients had a family history of CRC. A more detailed account of the cohort including smoking habits and body-mass index (BMI) is provided in Table 1.

**Table 1:** Prevalence of POLE EDMs and associations with clinical factors

	POLE mutation			Mutation in…			
		
	No	Yes	*P*-value	Exon 9	Exon 11	Exon 13	Exon 14
All patients	322 (87%)	46 (13%)	–	11 (3%)	7 (2%)	20 (5%)	11 (3%)
Age <70	174 (87%)	27 (13%)		9 (4%)	4 (2%)	11 (5%)	5 (2%)
Age 70+	148 (89%)	19 (11%)	0.55	2 (1%)	3 (2%)	9 (5%)	6 (4%)
Female	125 (89%)	15 (11%)		4 (3%)	3 (2%)	6 (4%)	3 (2%)
Male	197 (86%)	31 (14%)	0.42	7 (3%)	4 (2%)	14 (6%)	8 (4%)
Stage I	55 (83%)	11 (17%)		2 (3%)	1 (2%)	4 (6%)	5 (8%)
Stage II	105 (89%)	13 (11%)		3 (3%)	4 (3%)	5 (4%)	2 (2%)
Stage III	116 (90%)	13 (10%)		2 (2%)	1 (1%)	7 (6%)	3 (2%)
Stage IV	46 (84%)	9 (16%)	0.44	4 (7%)	1 (2%)	4 (7%)	1 (2%)
Grade 1 + 2	187 (87%)	29 (13%)		8 (4%)	3 (1%)	11 (5%)	8 (4%)
Grade 3 + 4	85 (87%)	13 (13%)	0.97	2 (2%)	4 (4%)	6 (6%)	3 (3%)
Location^1^							
Colon, proximal	85 (87%)	13 (13%)		5 (5%)	3 (3%)	5 (5%)	2 (2%)
Colon, distal	94 (89%)	12 (11%)		3 (3%)	3 (3%)	5 (5%)	2 (2%)
Rectum	140 (87%)	21 (13%)	0.89	3 (2%)	1 (1%)	10 (6%)	7 (4%)
BMI^2^							
<25 kg/m^2^	106 (89%)	13 (11%)		5 (4%)	1 (1%)	5 (4%)	2 (2%)
25–<30 kg/m^2^	147 (87%)	22 (13%)		5 (3%)	4 (2%)	9 (5%)	5 (3%)
30+ kg/m^2^	62 (89%)	8 (11%)	0.85	1 (1%)	1 (1%)	5 (7%)	2 (3%)
Nonsmoking	150 (88%)	21 (12%)		5 (3%)	4 (2%)	8 (5%)	5 (3%)
Former smoking	109 (88%)	15 (12%)		3 (2%)	1 (1%)	9 (7%)	3 (2%)
Current smoking	61 (86%)	10 (14%)	0.91	3 (4%)	2 (3%)	3 (4%)	3 (4%)
Family history CRC							
Yes	50 (96%)	2 (4%)		0 (0%)	0 (0%)	2 (4%)	0 (0%)
No/unknown	270 (86%)	44 (14%)	0.04	11 (4%)	7 (2%)	18 (6%)	11 (4%)
KRAS, wildtype	220 (88%)	30 (12%)		9 (4%)	4 (2%)	13 (5%)	6 (2%)
KRAS, mutation	80 (84%)	15 (16%)	0.32	2 (2%)	3 (3%)	7 (7%)	4 (4%)
KRAS, missing	21	1					
BRAF, wildtype	287 (87%)	41 (13%)		10 (3%)	7 (2%)	18 (5%)	9 (3%)
BRAF, mutation	24 (89%)	3 (11%)	0.98	0 (0%)	0 (%)	1 (4%)	2 (7%)
BRAF, missing	7	2					
CIMP low/neg	309 (88%)	43 (12%)		10 (3%)	7 (2%)	19 (5%)	10 (3%)
CIMP high	13 (81%)	3 (19%)	0.44	1 (6%)	0 (0%)	1 (6%)	1 (6%)

1 Distal colon from splenic flexure to sigmoid colon, rectum includes rectosigmoid.

2 BMI on average 10 years prior to diagnosis (range 5–14 years).

In total, 373 cases were analyzed for somatic mutations in the proofreading (exonuclease) domain of *POLE*. To this end, we sequenced exons 9, 11, 13, and 14 that correspond to amino acids 268–303, 341–369, and 410–491 of the exonuclease domain, respectively, thereby broadly covering the reported mutation hotspots at positions 286, 367, 411, and 459 [25].

**Figure 1:**
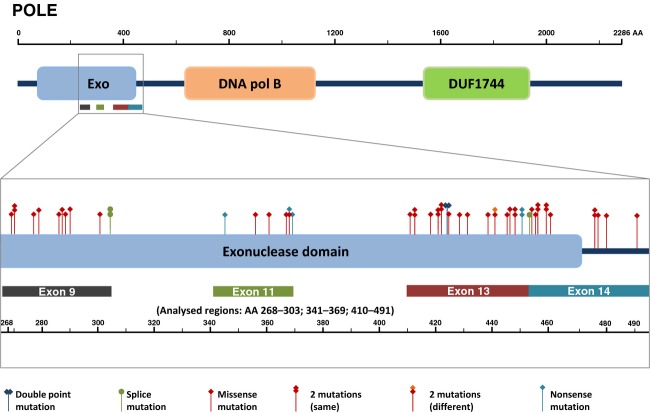
Distribution of POLE mutations within the exonuclease domain.

Overall we found 54, partly recurrent, mutations in 46 samples (out of 373, 12.3%) most of which have not been previously reported. The distribution of EDMs in POLE is depicted in Figure 1 and a detailed account is provided in Table 2: we detected 12 EDMs in exon 9 (A), 7 EDMs in exon 11 (B), 23 EDMs in exon 13 (C), and 12 EDMs in exon 14 (D).

**Table 2:** Detailed account of POLE EDMs per exon. (A) Exon 9, (B) Exon 11, (C) Exon 13, (D) Exon 14

cDNA	Amino acid	Frequency	COSMIC	MutationTaster	PolyPhen	PROVEAN	SIFT
(A) Exon 9							
c.805C>T	p.P269S	1		Disease causing	Probably damaging	Deleterious	Not tolerated
c.808G>A	p.V270M	2		Disease causing	Probably damaging	*Neutral*	Not tolerated
c.829G>A	p.E277K	1		Disease causing	Probably damaging	Deleterious	Not tolerated
c.836C>T	p.T279I	1		Disease causing	Probably damaging	Deleterious	Not tolerated
c.857C>T	p.P286L	1		Disease causing	Probably damaging	Deleterious	Not tolerated
c.859G>A	p.D287N	1		Disease causing	Probably damaging	Deleterious	Not tolerated
c.862G>A	p.A288T	1		Disease causing	*Possibly damaging*	*Neutral*	*Tolerated*
c.869C>T	T290I	1		Disease causing	*Benign*	Deleterious	*Tolerated*
c.901G>A	p.D301N	1		Disease causing	Probably damaging	Deleterious	Not tolerated
c.909 + 1G>A	p.splice?	2		Splice defect?			
(B) Exon 11							
c.1033C>T	p.Q345*	1		Truncating			
c.1066C>T	p.P356S	1		Disease causing	Probably damaging	Deleterious	Not tolerated
c.1082C>T	p.T361I	1		Disease causing	Probably damaging	Deleterious	Not tolerated
c.1099T>G	p.F367V	1		Disease causing	Probably damaging	Deleterious	Not tolerated
c.1102insT	p.D368*	1		Truncating			
c.1102G>A	p.D368N	1		Disease causing	Probably damaging	Deleterious	Not tolerated
c.1106G>A	p.W369*	1		Truncating			
(C) Exon 13							
c.1231G>A	p.V411M	1		Disease causing	Probably damaging	Deleterious	Not tolerated
c.1238G>A	p.R413K	2		Disease causing	Probably damaging	Deleterious	Not tolerated
c.1252C>T	p.P418S	1		Disease causing	Probably damaging	Deleterious	Not tolerated
c.1261A>G	p.S421G	1		Disease causing	Probably damaging	Deleterious	Not tolerated
c.1264C>T	p.H422Y	3		Disease causing	Probably damaging	*Neutral*	*Tolerated*
c.1269T>A	p.N423K	1		Disease causing	Probably damaging	Deleterious	Not tolerated
c.1270C>G	p.L424V	1		Disease causing	Probably damaging	Deleterious	Not tolerated
c.1270C>T	p.L424F	1		Disease causing	Probably damaging	Deleterious	Not tolerated
c.1283C>T	p.A428V	1		Disease causing	*Possibly damaging*	Deleterious	Not tolerated
c.1295T>C	p.L432P	1		Disease causing	Probably damaging	Deleterious	Not tolerated
c.1312G>C	p.E438Q	1		Disease causing	Probably damaging	Deleterious	Not tolerated
c.1321C>T	p.P441S	1		Disease causing	Probably damaging	Deleterious	Not tolerated
c.1322C>T	p.P441L	1		Disease causing	Probably damaging	Deleterious	Not tolerated
c.1334G>A	p.C445Y	1		Disease causing	Probably damaging	Deleterious	*Tolerated*
c.1337G>C	p.R446P	1		Disease causing	*Benign*	Deleterious	*Tolerated*
c.1342G>A	p.A448T	2		Disease causing	Probably damaging	Deleterious	Not tolerated
c.1351C>T	p.Q451*	2		Truncating			
c.1359 + 2C>T	p.splice?	1		Splice defect?			
(D) Exon 14							
c.1361C>T	p.T454I	1		Disease causing	*Benign*	*Neutral*	*Tolerated*
c.1366G>C	p.A456P	1	COSM 937318	Disease causing	Probably damaging	Deleterious	Not tolerated
c.1370C>T	p.T457M	2		Disease causing	*Possibly damaging*	Deleterious	*Tolerated*
c.1376C>T	p.S459F	2	COSM 170809	Disease causing	Probably damaging	Deleterious	Not tolerated
c.1382C>T	p.S461L	1		Disease causing	Probably damaging	Deleterious	Not tolerated
c.1427C>T	p.P476L	2		Disease causing	*Possibly damaging*	Deleterious	Not tolerated
c.1430T>C	p.F477S	1		Disease causing	Probably damaging	Deleterious	Not tolerated
c.1439C>T	p.A480V	1		Disease causing	Probably damaging	Deleterious	Not tolerated
c.1472A>G	p.E491G	1		Disease causing	*Possibly damaging*	Deleterious	Not tolerated

Italic indicates the important terms.

While missense mutations were most frequent (85.2%), sequencing also revealed truncating mutations in five cases, namely p.D368*, p.Q345*, p.W369* (all exon 11) as well as a recurrent p.Q451* mutation (9.2%) and three putative splice site mutations (1× c.1359 + 2C>T and 2× c.909 + 1G>A) (5.6%).

Interestingly, for six cases two different EDMs in each tumor have been detected. For these cases it remains unknown whether these mutations are located on the same allele or on different alleles. Two tumors harbored double mutations each within the same exon: exon 13 (p.N423K + p.K424V) and exon 14 (p.S459F + p.P476L), respectively. Another case showed a p.T279I mutation (exon 9) and a mutation of the splice-donor site of intron 9 (c.909 + 1). One tumor showed a double mutation in exons 13 and 14 (p.S421G and p.T457M) and two tumors displayed either double mutations (p.W369* and p.A480V) in exons 11 and 14, respectively, or double mutations (p.P356S and p.V270M) in exons 11 and 9, respectively. Moreover, two further cases harbored homozygous EDMs (or deletions of the second allele [LOH]), one with a p.R413K change (exon 13) and one with a p.H422Y (exon 13) change, respectively.

In accord with previous data [9–11], two cases revealed a known p.S459F mutation and we detected a p.V411, a p.P286 and a p.F367 mutation in one case each. For each of the latter three EDMs, however, we found different amino acid substitutions in contrast to what has been reported previously: for codon 411, we identified a methionine substitute instead of leucine, for codon 286 we observed a leucine substitution instead of arginine, and for residue 367 we found phenylalanine replaced by valine instead of serine. Of note, we detected a point mutation in exon 9 leading to amino acid substitution of glutamic acid by lysine at codon 277, which is an active site within the conserved exo I motif (residues 271–285) required for exonuclease function. We also found two mutations in codon 424 with a classic p.L424V, which has already been reported as germline mutation [8] and a further mutation showing substitution by phenylalanine. Two cases displayed a p.V270M mutation each, which has already been determined as germline SNP by the NHLBI exome sequencing project (rs374237142, present in one of 6503 genotypes; http://evs.gs.washington.edu/EVS/). Moreover, we found a p.A456P mutation that has already been annotated in COSMIC suggesting a recurrent somatic aberration.

To estimate the biological implications of the sequencing data in silico, we applied four different software tools that allow for the prediction of the deleteriousness on protein function of each somatic mutation and found ∼75% of the mutations classified as harmful by all four algorithms. This rate was considerably higher (almost 100%) when cases were included for which at least one software tool predicted a negative effect on protein function (for details see Table 2).

### Associations of POLE mutations with clinical parameters

To determine whether POLE EDMs in MSS CRC constitute a tumor type with specific clinical characteristics, we investigated the associations of POLE mutations with major clinical parameters. As depicted in detail in Table 1, we neither found associations with age and sex nor with tumor-specific measures including tumor stage and grade as well as tumor location. Also, BMI and smoking habits, both of which have been implicated in CRC tumorigenesis were not found to be associated with POLE EDMs. Notably, we could not determine an association between POLE mutations and a positive family history of CRC. Associations between BRAF mutations or the CIMP-phenotype were not observed. We also did not see any associations with the mutational status of KRAS.

### Survival analysis

Next, we investigated whether POLE EDMs have an impact on OS and DSS of MSS CRC patients.

**Figure 2:**
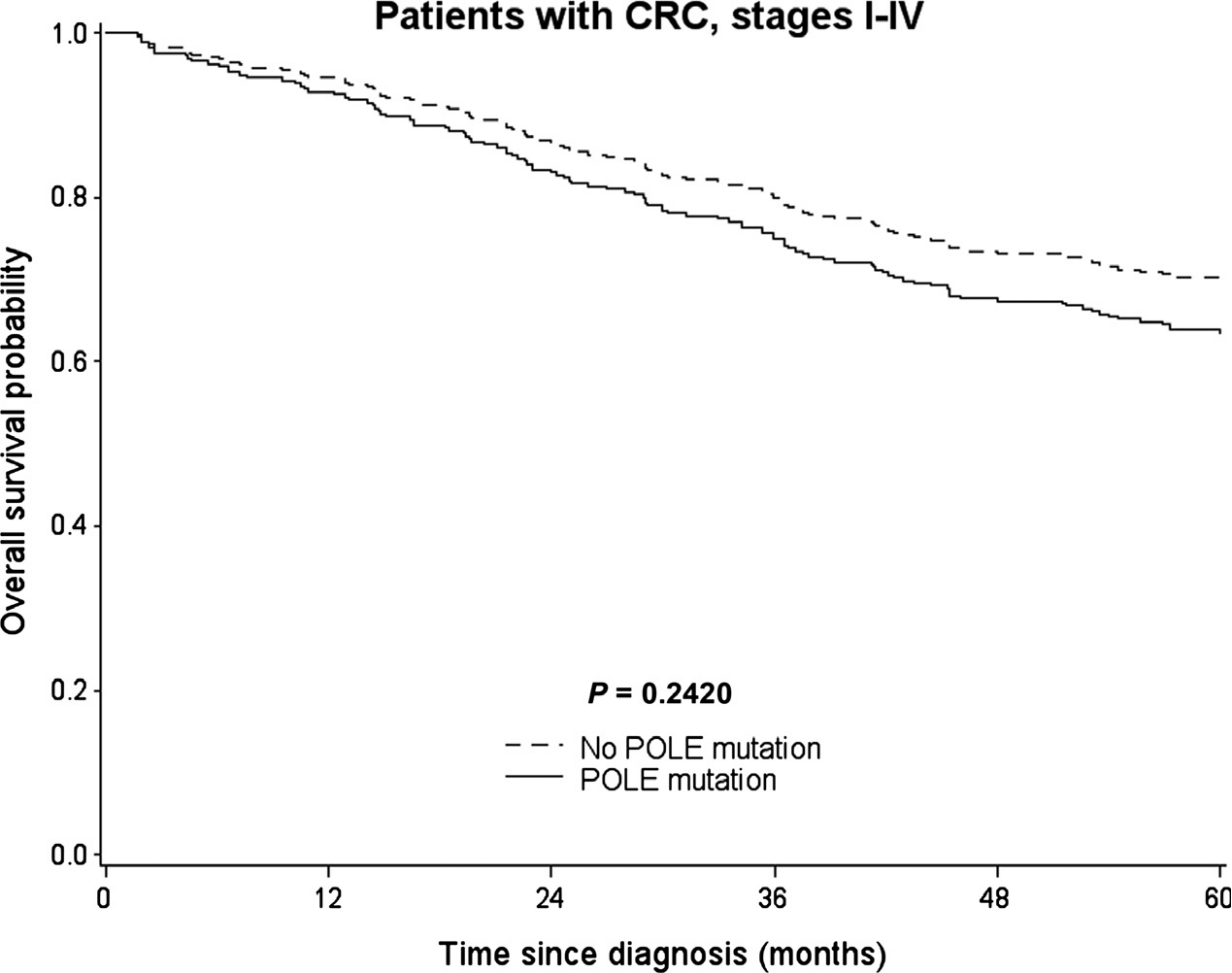
Direct adjusted survival curves for the association of POLE mutation and overall survival among patients with colorectal cancer (CRC), stages I-IV. Survival curves are adjusted for age, sex, CRC stage, CRC location, chemotherapy, and neoadjuvant treatment.

**Figure 3:**
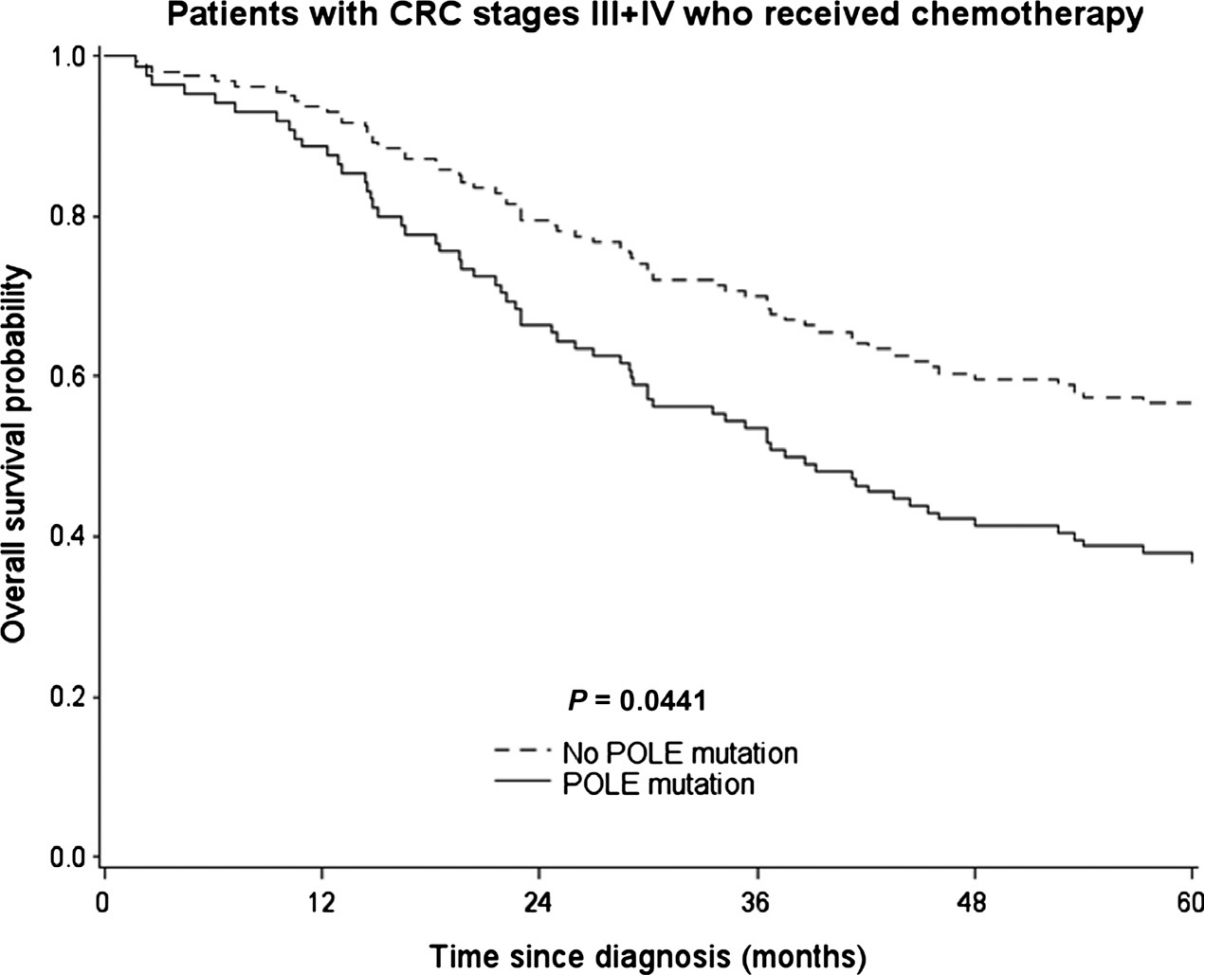
Direct adjusted survival curves for the association of POLE mutation and overall survival among patients with colorectal cancer (CRC), stages III+IV, who received chemotherapy. Survival curves are adjusted for age, sex, CRC stage, CRC location, and neoadjuvant treatment.

In the overall cohort, POLE EDMs were found to confer a slightly higher risk for impaired outcome compared to POLE wildtype cases (adj HR: 1.35, 95% CI: 0.82–2.25) but did not prove to be statistically significant (Table 3). Correspondingly, adjusted survival curves (accounting for the effect of all major confounding covariables) revealed no statistically significant difference in OS between patients with POLE mutated and those with POLE wildtype tumors (*P* = 0.24; Fig. 2). In line with this finding, stratification for different types of mutations and mutational subgroups generally revealed slightly increased HRs (for details see Table 3). However, these results were not statistically significant. When zooming in on different clinical subgroups (Table 4), we observed different hazard ratios for patients with POLE-mutated tumors with respect to sex, age, grade, and location of tumor, all of which were, again, not statistically significantly different. However, when looking at patients across all disease stages who received chemotherapy, we observed an increased adjusted hazard ratio of 1.82 (95% CI: 0.99–3.34) and focused analysis of patients with stage III/IV disease who received either adjuvant or palliative chemotherapy revealed statistically significantly increased mortality for patients with POLE-mutated CRCs (adj. HR: 1.87; 95% CI: 1.02–3.44). This finding is further illustrated by the results of the direct adjusted survival analysis depicted in Figure 3.

**Table 3:** Association of POLE mutational subgroups with overall survival

			Overall survival	
			
	*N*	All deaths	Unadjusted HR (95% CI)	Adjusted HR (95% CI)^1^
No POLE mutation	322	109 (34%)	1.00 Ref	1.00 Ref
POLE mutation	46	18 (39%)	1.14 (0.69–1.88)	1.35 (0.82–2.25)
Exon 9 mutation	11	5 (45%)	1.40 (0.57–3.44)	1.37 (0.54–3.47)
Exon 11 mutation	7	2 (29%)	0.78 (0.19–3.17)	0.98 (0.24–4.04)
Exon 13 mutation	20	7 (35%)	1.04 (0.49–2.24)	1.21 (0.56–2.62)
Exon 14 mutation	11	5 (45%)	1.30 (0.53–3.18)	1.78 (0.71–4.49)
Missense type	40	15 (38%)	1.09 (0.64–1.88)	1.41 (0.81–2.43)
Splice type	3	2 (67%)	1.77 (0.44–7.16)	1.45 (0.35–6.11)
Truncating type	5	1 (20%)	0.54 (0.08–3.84)	0.44 (0.06–3.18)
Known codon forgermline mutation	4	1 (25%)	0.71 (0.10–5.09)	0.58 (0.08–4.21)
Known codon forsomatic mutation	4	1 (25%)	0.54 (0.08–3.86)	1.03 (0.14–7.65)
Double mutations	8	1 (13%)	0.33 (0.05–2.34)	0.43 (0.06–3.08)
Somatic mutations^3^	42	17 (40%)	1.19 (0.71–1.98)	1.47 (0.87–2.48)
Harmful mutation^2^	40	15 (38%)	1.06 (0.62–1.81)	1.26 (0.73–2.18)
Nonharmful mutation^2^	6	3 (50%)	1.88 (0.60–5.92)	2.27 (0.70–7.39)

Statistical analysis accounts for late entry, that is, the potentially delayed time period between date of diagnosis and date of interview.

1 Adjusted for age, sex, stage at diagnosis, location of colorectal cancer (proximal colon/distal colon/rectum), adjuvant and neoadjuvant therapy.

2 As predicted by in silico analyses.

3 Putative germline mutations (as reported in the current literature) excluded.

**Table 4:** Association of POLE EDMs with overall survival in clinical subgroups

	POLE mutation		No POLE mutation		Overall survival	
	*N*	All deaths	*N*	All Deaths	Unadjusted HR (95% CI)	Adjusted HR (95% CI)^1^
<70 years	27	9 (33%)	174	44 (25%)	1.41 (0.69–2.88)	1.25 (0.60–2.63)
≥70 years	19	9 (47%)	148	65 (44%)	0.95 (0.47–1.91)	1.72 (0.79–3.76)
Female	15	5 (33%)	125	55 (44%)	0.82 (0.33–2.04)	0.92 (0.36–2.35)
Male	31	13 (42%)	197	54 (27%)	1.48 (0.81–2.71)	1.65 (0.89–3.07)
Stages I+II	24	4 (17%)	160	29 (18%)	0.83 (0.29–2.36)	0.75 (0.26–2.19)
Stages III+IV	22	14 (64%)	162	80 (49%)	1.42 (0.80–2.50)	1.54 (0.85–2.78)
Grade 1 + 2	29	11 (38%)	187	54 (29%)	1.34 (0.70–2.57)	1.87 (0.95–3.67)
Grade 3 + 4	13	7 (54%)	85	38 (45%)	1.14 (0.51–2.56)	1.14 (0.49–2.70)
Colon, proximal	13	6 (46%)	85	34 (40%)	1.17 (0.49–2.80)	1.51 (0.62–3.73)
Colon, distal	12	4 (33%)	94	27 (29%)	1.05 (0.37–3.01)	1.24 (0.42–3.71)
Rectum	21	8 (38%)	140	45 (32%)	1.19 (0.56–2.52)	1.44 (0.65–3.20)
Chemotherapy, all stages	20	14 (70%)	129	60 (47%)	1.84 (1.03–3.31)	1.82 (0.99–3.34)
No chemotherapy, all stages	26	4 (15%)	192	48 (25%)	0.54 (0.19–1.50)	0.69 (0.24–1.96)
Chemotherapy, stages III+IV	19	14 (74%)	118	58 (49%)	1.84 (1.03–3.32)	1.87 (1.02–3.44)

Statistical analysis accounts for late entry, that is, the potentially delayed time period between date of diagnosis and date of interview.

1 Adjusted for age, sex, stage at diagnosis, location of colorectal cancer (proximal colon/distal colon/rectum), adjuvant and neoadjuvant therapy.

Results for the associations of POLE mutations with DSS (adj HR: 1.44, 95% CI: 0.81–2.58) were very similar to the results on OS, also in the subgroups. However, subgroup analyses were limited by the lower number of events of disease-specific survival analyses (data not shown).

## Discussion

Several decades of research into CRC have revealed that on biological grounds, CRC cannot be viewed as one cancer entity but comprises distinct molecular tumor subtypes, which are each associated with a specific clinical behavior with implications for oncological therapy [26].

While the three major biological CRC phenotypes CIN, MSI, and CIMP and their clinical implications have been elucidated during the last 20 years, it was only recently noted that a subset of MSS CRC patients harbor germline mutations in the exonuclease domain of POLD and POLE [8], which account for the exonuclease catalytic activities of these DNA polymerases [27]. Germline mutations of POLD and POLE were shown to predispose individuals to a polyposis-phenotype with large adenomas similar to that observed in MUTYH-associated polyposis or early onset and multilocated cancers, respectively [8]. Also recently, two other studies independently discovered that recurrent somatic EDMs of POLE occur in approximately 3% of CRC [9, 10]. Both, germline and somatic mutations were exclusively found in MSS CRC and were associated with a so-called “ultramutator phenotype”, even exceeding mutation rates observed in MSI tumors. These data argue for a unique biological subtype of CRC whose clinical properties have not yet been elucidated.

Very interestingly, two additional studies [28, 29] employing next-generation sequencing approaches also very recently reported on the presence of EDMs of POLE in endometroid endometrial carcinomas (EC) at a slightly higher frequency (around 7%) than observed for CRC. Again, these mutations were found to be associated with the above mentioned ultramutator phenotype strongly suggesting a causal relationship between the loss of function of the DNA polymerase and the mutation frequency of the tumor. In addition, the study on EC conducted by the TCGA [28], although in a very exploratory manner, addressed the question whether POLE mutations have a clinical impact and found POLE mutated tumors to be associated with an exceptionally good prognosis compared to the other molecular subgroups of EC. It has been hypothesized that this favorable outcome may be explained by the fact that with their extreme genetic instability POLE mutated tumors are unable to cope with DNA damage induced by cytotoxic treatment. However, these data are preliminary and the observations were based on a small cohort, with limited clinical annotations. Despite these limitations, the current data on CRC and EC prompt the question if POLE EDMs in CRC may also have direct clinical implications and are related to a particular clinical phenotype.

By sequencing 373 MS-stable CRC of a population-based observational study, we identified a higher frequency of somatic POLE mutations compared to previous reports (12.3% vs. 3%). We mainly attribute these differences to a more sensitive mutation calling by conventional Sanger sequencing focusing on previously determined genomic hotspots compared to the explorative whole exome next-generation sequencing approaches [9, 10] using rather low read depth. Additionally, enrichment of MSS cases may at least partly contribute to the increased EDM frequency observed in our cohort. The majority of the mutations were of missense type and also included the four already reported recurrent somatic mutations leading to amino acid substitutions at positions 286, 367, 411, and 459. We identified several novel missense mutations as well as cases in which both alleles of POLE might be affected by mutations. Moreover, we discovered a recurrent truncating mutation, mutations hitting splice sites of POLE and two mutations affecting codon 424, which was previously reported to be affected by germline mutations. Very interestingly, we found a c.829G>A mutation in exon 9 leading to a p.E277K change on protein level. This residue is part of the conserved exo I motif (residues 271–285) required for exonuclease function. To our knowledge, this is the second (the other being p.D275V) missense mutation reported to directly alter a catalytic amino acid within this motif. As the EDMs of POLE have not yet been reported as nondisease associated germline variations in the respective large databases (e.g., dbSNP, exome variant server [EVS]), the herein detected mutations likely represent true disease relevant molecular alterations. This is supported by the fact that insilico analysis of the mutations predicted a negative biological impact on the corresponding protein function for the majority of mutations.

In contrast to our assumption, we did not observe any significant associations of POLE mutations in general as well as mutation subgroups with major epidemiological clinical and genetic parameters in the total cohort. Overall, we recognized an increased hazard for patients with POLE mutated CRCs, which, however, did not reach statistical significance. These findings stand in contrast to the results reported for EC [29] and remain to be corroborated by independent studies as other data are currently not available on this issue. Our population-based study cohort of MSS CRC is of considerable size and thoroughly characterized, but we cannot exclude that with even larger studies a putative adverse effect of POLE EDMs would become more obvious and statistically significant. Pointing to this direction, the results for patients with stage III/IV tumors receiving adjuvant or palliative chemotherapy according to the German treatment guidelines of the observation period (2003–2006) demonstrate that patients in this subgroup harboring POLE mutated tumors have a statistically significantly increased mortality. Hence, it is tempting to speculate and remains to be investigated in further studies whether EDMs in POLE have prognostic or predictive implications in these patients and if this were true, to unravel the underlying biological mechanism.

In this context, it is important to note that we have used a cohort derived from an epidemiological study rather than from a clinical trial. The reason for this was our assumption that a broad approach using a cohort that reflects an average CRC patient population should potentially uncover relations between POLE EDMs and clinicopathological parameters of CRCs if the biological impact of EDMs on a particular CRC phenotype is strong. While we acknowledge the fact that a cohort derived from a controlled epidemiological study is clinically more heterogeneous than a well-designed phase III trial, we would like to emphasize that (1) the calculated hazard ratios and Kaplan–Meier curves have been adjusted for all major potentially confounding factors including age, sex, stage at diagnosis, location of the tumor and different therapy regimens and (2) we aimed at achieving a fairly homogenous patient cohort of which all patients have been treated at the University Hospital Heidelberg according to the established guidelines. Interestingly, the calculated adjusted and unadjusted hazard ratios differ only slighty indicating rather low influence by putative confounders. Given the currently limited knowledge on the precise role of POLE EDMs and their clinical implications in CRCs, it may be worthwhile to consider that the analysis of a particular clinical trial designed to measure the outcome of a particular therapy by specific endpoints in a highly selected patient cohort might prematurely have narrowed the perspective thereby potentially introducing a bias.

Since the precise functional role of POLE aberrations in cancer development and specifically in CRC has not yet been fully understood, a satisfactory biological explanation of our results is challenging. However, it is tempting to speculate that in humans (1) the degree of biological impact of somatic POLE aberrations on protein function and in turn clinical relevance appear to be cancer-specific rather than of general and equal importance and (2) a somatic mutation in one allele of POLE per se may not necessarily be sufficient to yield a specific clinically distinguishable phenotype. The latter assumption is in line with the observation that only mice homozygous for mutant POLE develop a mutator phenotype accompanied by increased frequencies of tumor formation [16]. This finding may be attributable to a great redundancy of evolutionary conserved repair systems to maintain DNA integrity throughout life [30] and may also depend on a heterogeneous impact of each type of mutation on protein function, which has not been explored in vivo yet. Our data also suggest that even tumors with aberrations in both alleles of POLE (‘double hit’-phenotype) or double mutations of POLE do not necessarily differ from counterparts with wildtype alleles or with a mutation in one allele with respect to clinical features of the tumor. However, our cohort comprised only very few of those cases and is therefore of limited informative value. As suggested by our data, EDMs may play a crucial role in specific clinical subgroups of CRC. As we show here, EDMs in advanced tumors, which have already metastasized seem to interfere with response to chemotherapy and are associated with dismal prognosis. The reason for this is currently unclear and it is tempting to speculate whether certain chemotherapeutic agents add to the adverse effect of mutated POLE on DNA integrity by enhancing the likelihood to gain additional genetic aberrations which in turn may confer a more malignant genotype and subsequent phenotype stochastically. In light of our data, it would certainly be of interest to gain deeper understanding of the interplay between functionally impaired polymerase *ε* and drugs used in the, for example, folinic acid-leucovorin-oxaliplatin regimen.

It should be noted that the conclusions presented here do not contradict a functional relevance of EDMs in POLE for carcinogenesis and predisposition to CRC as has for example been shown for germline mutations by Palles et al. [8].

To conclude, we show that the frequency of POLE mutations in MSS CRC is considerably higher than previously reported including splice-site, truncating and double mutations and provide evidence that albeit biologically different from the other molecular subtypes, POLE mutated CRCs in general do not appear to constitute an entirely new entity from the clinical viewpoint, since they lacks specific features that allow for a separation of these tumors from the whole class of CRC in terms of epidemiology and outcome. It remains to be investigated, however, if EDMs in POLE have prognostic or predictive implications in patient subgroups such as stage III/IV disease treated with chemotherapy.
